# Urinary Tract Infection and Fetal Outcomes Among Pregnant Women in Adare General Hospital, Hawassa, Ethiopia

**DOI:** 10.1155/ijm/8562296

**Published:** 2024-12-19

**Authors:** Ararso Agegnehu Yetera, Tadesse Menjetta Nima, Musa Mohammed Ali, Moges Desta Ormago

**Affiliations:** ^1^Department of Laboratory, Adare General Hospital, Hawassa, Ethiopia; ^2^School of Medical Laboratory Science, College of Medicine and Health Sciences, Hawassa University, Hawassa, Ethiopia

**Keywords:** drug resistant, poor fetal outcomes, pregnant women, UTI

## Abstract

**Background:** Fetal complications can occur if pregnant women with urinary tract infection (UTI) are not treated. We aimed to determine the magnitude of UTI, drug resistance profile, and fetal outcomes among pregnant women in Adare General Hospital, Hawassa, Ethiopia.

**Methods:** Facility-based cross-sectional study was conducted among 308 pregnant women using questionnaire and review of medical records. From 308 randomly selected pregnant women, clean catch midstream urine samples were collected, processed, and inoculated onto MacConkey and blood agars and after incubation, the colonies were further confirmed by using standard biochemical tests. A binary logistic regression model was used to compute the explanatory variables with the outcome variable. A *p* value less than 0.05 was considered statistically significant.

**Results:** The overall prevalence of UTI was 13.6% with a 95% CI: 10–18. Out of 42 samples, 39 (92.8%) UTI infections in women between the ages of 15 and 34 were identified. The three most common bacterial isolates were *Escherichia coli*, *Staphylococcus aureus*, and *Staphylococcus saprophyticus*. The majority of the Gram-negative bacteria isolates were resistant to ampicillin (96.2%) and trimethoprim-sulfamethoxazole (39%), while the Gram-positive bacteria were resistant to tetracycline (75%) and trimethoprim-sulfamethoxazole (68.8%). Of the total 308 pregnant women who participated in the study, there were 51 (16.6%) poor fetal outcomes. In this study, the presence of bacteriuria had a significant association with poor fetal outcomes (*p* value = 0.001). The mother's age, gravidity, level of education, occupation, marital status, and previous UTI history were not associated with the current UTI status.

**Conclusions:** Poor fetal outcomes are strongly associated with UTI during pregnancy. Early detection of UTI and treatment after culture and antibiotic susceptibility testing should be a priority for the management of UTIs in pregnancy to avoid poor fetal outcomes.

## 1. Background

Urinary tract infections (UTIs) are the most common bacterial infections during pregnancy and account for about 10% of women's hospital visits. It is a leading cause of maternal and newborn morbidity and mortality during pregnancy and is associated with different maternal and neonatal adverse outcomes such as low birth weight, preterm birth, stillbirth, preeclampsia, maternal anemia, sepsis, and amnionitis, even when the infection is asymptomatic [1–3]. Due to the short urethra of women, which open near the anus, the nature of sexual activity, pregnancy, and simple contamination of the urinary tract by fecal flora, and the rapid development of hormonal changes, women are three times more likely to develop UTI than men [4]. To avoid complications, pregnant women should be checked for asymptomatic UTI and treated with suitable antimicrobials [5]. If left untreated, UTI can cause serious complications such as poor maternal and neonatal outcomes.

As a result, this study aimed to determine the magnitude of UTIs, antibiotic resistance patterns and associated fetal problems in pregnant women attending the antenatal care clinic at Adare General Hospital in Hawassa. This study gives information for clinicians and hospital management for selection of drugs for treatment UTI in pregnant women who are visiting their facility.

## 2. Materials and Methods

### 2.1. Study Area and Period

The study was conducted at Adare General Hospital in Hawassa City from May to August 2022. Hawassa is the capital city of the Sidama region and is located 275 km from Ethiopia's capital city, Addis Ababa. The town is located 1697 m above the sea level, with mean annual temperatures of 20.9°C and rainfall of 997.6 mm. According to the population census in 2007 E.C., the total population of the city was 386,773.

Adare General Hospital serves a catchment population of 1,368,341. Every day, more than 74 pregnant women visit an ANC clinic; more than eighteen of them are in the third trimester. The hospital had 250 beds with an average bed occupancy rate of 85%. More than 90% of pregnant women gave birth at Adare General Hospital attending ANC follow-up. The remaining 8.44% of pregnant women did not give birth at Adare General Hospital after their follow-up at ANC Clinic.

### 2.2. Study Design and Population

A hospital-based cross-sectional study was conducted among third-trimester pregnant women who attended an antenatal clinic at Adare General Hospital and fulfilled the inclusion criteria from May to August 2022. Pregnant women in their third trimester and who attended and gave consent to give birth at Adare general Hospital during the study period were included in the study whereas pregnant women who were receiving antibiotics during the past 14 days of data collection period were excluded from the study. Only pregnant women at third trimester were enrolled to minimize the waiting time to assess fetal outcome.

### 2.3. Sample Size Determination and Sampling Techniques

The sample size was estimated using a single population proportion formula (*n* = *z*^2^*p*[1 − *p*]/*d*^2^) considering a 95% level of confidence and a 5% margin of error, 10% anticipated nonresponse rate, and prevalence of 23.9% reported from Madda Walabu University Goba Referral Hospital, Bale-Goba, Ethiopia [6]. Thus, the total sample size calculated was 308. The participants were selected by using a systematic random sampling technique. Considering the study period, 1320 pregnant women in their third trimester of gestational age were expected to visit the clinic during the study period as per a four-month activity report and plan of action. The estimated population was divided by sample size (1320/308) to determine the *k* value which was four. The first participant was selected by lottery method and then followed by a selection of every fourth of the study participants until the required sample size was achieved.

### 2.4. Sociodemographic and Obstetrics Data

Semistructured questionnaire was used to collect the participant's age, marital status, and educational status, and ANC log book was used to collect UTI signs and symptoms and fetal outcomes such as appearance, pulse, grimace, activity, and respiration (APGAR) scores in 5 min, birth weight, premature rupture of membrane (PROM), preterm labor, or intra uterine fetal death (IUFD).

### 2.5. Specimen Collection

Clean catch midstream urine samples were collected in sterile, dry, wide-necked, leak-proof, screw-capped containers from study participants who were attending the antenatal clinic at Adare General Hospital from May to August 2022. The samples were transported to the Hawassa University School of Medical Laboratory within an hour of collection.

### 2.6. Cultivation and Identification of Isolates

A calibrated sterile wire loop was used to inoculate 1 μL of urine specimen on MacConkey agar (Oxoid, Hampshire, UK) and blood agar (Oxoid, Hampshire, UK). Colonies were counted to check significant bacteriuria (> 10^5^ bacterial counts per mL of urine) after 24 h of incubation at 37°C. The isolated bacterial pathogens were identified using colony characteristics, gram staining reaction, and profile of biochemical reaction using the standard microbiological techniques. Enterobacteriaceae were identified by H_2_S production and carbohydrate fermentation in KIA agar, indole production, citrate utilization, motility test, urease test, and lysin decarboxylation test. The Gram-positive cocci were identified using catalase and coagulase tests [7, 8].

### 2.7. Antimicrobial Susceptibility Testing

Antimicrobial susceptibility testing was performed on isolates using the Kirby–Bauer disc diffusion method on Muller–Hinton agar following the Clinical and Laboratory Standards Institute criteria [9]. A loopful of pure bacterial colonies was mixed in 5 mL of 0.85% saline solution to make a homogeneous bacterial suspension. After adjusting the suspension to 0.5 McFarland, it was evenly inoculated on Muller–Hinton agar plate with sterile cotton swab and antibiotic disks at 15 mm and 24 mm distances from the edge and from each other, respectively. After that, the plate was incubated for 24 h at 37°C. Based on the inhibition zone produced around the antibiotics used, the isolates were classified as sensitive (S), resistant (R), or intermediate (I).

Ampicillin (10 μg), ampicillin/sublactam (10 μg), piperacillin/tazobactam (10 μg), cefazolin (10 µ), cefoxitin (30 μg), ceftazidime (30 μg), ceftriaxone (30 μg), cefepime (30 μg), meropenem (10 μg), gentamicin (10 μg), tobramycin (30 μg), ciprofloxacin (5 μg), and trimethoprim/sulfamethoxazole (23.75 μg) were used for susceptibility test of Gram-negative bacteria.

Amoxicillin (20 μg), penicillin (10 units), cefotaxime (30 μg), ceftriaxone (30 μg), chloramphenicol (30 μg), erythromycin (15 μg), ertapenem (10 μg), levofloxacin (5 μg), linezolid (10 μg), moxifloxacin (10 μg), meropenem (10 μg), ofloxacin (2 μg), tetracycline (30 μg), telithromycin (15 μg), trimethoprim/sulfamethoxazole (23.75 μg), and vancomycin (30 μg) were used for Gram-positive bacteria [9].

### 2.8. Data Quality Control

The questionnaire was pretested on 5% of pregnant mothers representing the total sample size to check its consistency before the actual data collection. Two BSc degree-holder midwives and two laboratory technologists were trained before data collection. Furthermore, SOPs of the microbiology laboratory were followed. Completeness, accuracy, and clarity of the filled questionnaire were checked daily. Before inoculation, the media's sterility was checked by overnight incubation at 37 degrees Celsius. To test the growth support of the media, quality control bacteria was used. Blood agar plate (*S. aureus* ATCC 25923, *E.coli* ATCC 25922, and *E. faecalis* ATCC 29212), MacConkey agar plate (*E.coli* ATCC 25922, *P. mirabilis* 12,453, and *E. faecalis* ATCC 29212), and Muller–Hinton agar plate (*S. aureus* ATCC 25923, *E.coli* ATCC 25922, and *E. faecalis* ATCC 29212) were used [9].

### 2.9. Data Processing and Analysis

All filled questionnaires for this study were checked visually, coded, and entered into Excel and then exported to SPSS Version 25 software (SPSS Inc., Chicago, IL, USA) for statistical analysis. The study's findings were explained further in text, words, and tables. Crude and adjusted odds ratios with 95% confidence intervals (CIs) were calculated using simple logistic regression analysis and multivariable logistic regression analysis, respectively, to assess the association between dependent and independent variables. Each variable with a *p* value of less than 0.25 in the simple logistic regression analysis was entered into the multivariable logistic regression analysis. A *p* value of < 0.05 is considered as statistically significant.

### 2.10. Operational Definition

UTI: a pregnant woman was said to have UTI when either of asymptomatic bacteriuria or symptomatic bacteriuria was present.

Asymptomatic UTI is a type of UTI that is diagnosed by urine culture test that yield significant (≥ 10^5^ cfu/mL) amount of similar single bacterial species from clean catch urine from an individual without symptoms of UTI.

Symptomatic UTI is a condition which is characterized by the presence of significant bacteriuria in midstream urine specimens that yielding positive cultures (≥ 10^5^ cfu/mL) with accompanying symptoms such as dysuria, urgency, frequency, incontinence, suprapubic pain, flank pain or costovertebral angle pain, tenderness, and fever (temp. ≥ 38°C).

Adverse fetal outcomes: the occurrence of one or more of the following fetal outcomes: LBW (delivery of a live infant whose birth weight was less than 2500 g), preterm (babies born alive before 37 weeks of pregnancy are completed), low Apgar score at 5 minutes (less than 7), neonatal death (death of a live birth within 28 days), and stillbirth (fetal death at or after 28 weeks gestation).

### 2.11. Ethics Approval and Informed Consent

Ethical clearance was obtained from Hawassa University College of Medicine and Health Science Institution Review Board (IRB) with Ref. no: IRB/274/12. A permission and support letter was obtained from Hawassa city health office. Informed written consent was obtained from all participants and also informed about the rights to terminate participating in the research at any time of the study. Confidentiality of information was maintained by codes and put in a lockable cabinet. Clinicians were communicated the findings for the management of patients. All methods were carried out in accordance with the relevant guidelines and regulations.

## 3. Results

### 3.1. Sociodemographic and Obstetric Characteristics

A total of 308 pregnant women participated during the study period. In this study, the mean age of the study participants was 24.7 ± 4.4 years old and within the age range of 15–39. Half of the study participants, 158 (51.3%), were 15–24 years old. The majority of the participants, 303 (98.4%), were married. Around 18% of the study participants had primary school level of education. One hundred and eighty three (59.4%) of the study's participants were housewives. Most study participants, 252 (81.8%), were multigravida, and nearly 7% and 1.6% of pregnant mothers presented with PROM and IUFD, respectively. Participants gave birth with 285 (92.5%) normal birth weight and 297 (96.4%) normal APGAR score and 303 (98.4%) gestational age at birth was term. Twenty seven (8.8%) of them had a history of frequent urination (Table 1).

### 3.2. Prevalence of UTI

The overall prevalence of UTI was 42 (13.6%) (95% CI: 10–18), of which 14 (4.5%) and 28 (9.1%) were symptomatic bacteruria and asymptomatic bacteruria, respectively. Ten different types of bacteria were isolated from urine samples of pregnant mothers with UTI. The majority of the isolates, 26 (61.9%), belong to Gram-negative bacteria. Among total isolates, the predominant bacteria were *E. coli* 17 (40.5%), followed by *S. aureus* 6 (14.3%), *S. saprophytics* 4 (9.5%), *K. pneumoniae* 3 (7.1%), *E. faecalis* 3 (7.1%), *S. pyogenes* 3 (7.1%), *K. oxytoca* 2 (4.8%), *Citrobacter* spp 2 (4.8%), *Proteus mirabilis* 1 (2.4%), and *M. morganii* 1 (2.4%) (Table 2).

### 3.3. Antibiotic Susceptibility Pattern

#### 3.3.1. Gram-Negative Bacteria

Gram-negative bacteria were resistant to ampicillin (96.2%), trimethoprim-sulfamethoxazole (39%), ampicillin/sublactam (34.6%), tobramycin (34.6%), ceftriaxone (30.7%), ceftazidime (23%), cefazolin (23%) and high rate of sensitive were also observed to cefoxitin (92%), cefepime (92%), and ciprofloxacin (85.5%).

*Escherichia coli* were highly resistant to ampicillin (94.1%), ampicillin/sulbactam (35.3%), and cefazolin (23.5%) but sensitive to cefepime (94.1%), meropenem (94.1%), tobramycin (94.1%), and cefoxitin (88.2%).

*K. pneumoniae* were highly resistant to ampicillin (100%) and 66.7% were resistant to cefazolin, ampicillin/sulbactam, and trimethoprim-sulfamethoxazole each but all *K. pneumoniae* isolates were sensitive to tobramycin, cefepime, gentamicin, meropenem, and ciprofloxacin. *K. oxytoca* was 100% resistant to ampicillin; meropenem, and ceftriaxone and 100% sensitive to cefazolin, cefoxitin, tobramycin, and ciprofloxacin each. *Citrobacter* spp were highly resistant to ampicillin (100%), ceftazidime (100%), trimethoprim-sulfamethoxazole (100%), and 100% sensitive were observed to tobramycin, cefazolin, cefoxitin, and ciprofloxacin for each. *Proteus mirabilis* was resistant to meropenem, gentamicin, ceftazidime, trimethoprim-sulfamethoxazole, and ampicillin to 100% for each and 100% sensitive to tobramycin, cefoxitin, ciprofloxacin, and piperacillin/tazobactam for each. *M. morganii* were resistant to ampicillin, ceftazidime, and trimethoprim-sulfamethoxazole but intermediate to gentamicin 100%. *M. morganii* were sensitive to cefepime, ampicillin/sublactam, piperacillin/tazobactam, cefazolin, and cefoxitin (Table 3).

#### 3.3.2. Gram-Positive Bacteria

Gram-positive isolates were highly sensitive to moxifloxacin (100%), ofloxacin (87.5%), linezolid (87.5%), meropenem (75%), and levofloxacin (75%) while resistant to tetracycline (75%), trimethoprim-sulfamethoxazole (68.8%), ceftriaxone (37.5%), cefotaxime (37.5), amoxicillin (31.3%), and penicillin (31.3%).

*S. aureus* was highly sensitive to linezolid (100%), moxifloxacin (100%), ofloxacin (83.3%), erythromycin (83.3%), ceftriaxone (83.3%), meropenem (83.3%), and amoxicillin (83.3%) but resistant to trimethoprim-sulfamethoxazole (66.7%) and tetracycline (66.7%) and 33.3% resistant to levofloxacin, telithromycin, and cefotaxime each.

*S. saprophytics* were highly sensitive to moxifloxacin (100%) and 75% to meropenem, amoxicillin, cefotaxime, chloramphenicol, linezolid, and ofloxacin for each while resistant to tetracycline (100%), trimethoprim-sulfamethoxazole (100%), ceftriaxone (75%), penicillin, ertapenem, vancomycin, and telithromycin 50% each.

All *E. faecalis* were sensitive to levofloxacin, linezolid, moxifloxacin, and ofloxacin, and *E. faecalis* were also sensitive to ceftriaxone (67%) and meropenem (67%) but highly resistant (67%) to trimethoprim-sulfamethoxazole, cefotaxime, and tetracycline each.

All *S. pyogenes* were sensitive to levofloxacin, chloramphenicol, ertapenem, moxifloxacin, meropenem, and ofloxacin, and *S. pyogenes* was sensitive to linezolid (67%) and ceftriaxone (67%) while resistant to amoxicillin (67%) and tetracycline (67%) (Table 4).

Among the total bacterial isolates (*n* = 42), the overall prevalence of multidrug resistance pattern was recorded as 28 (66.7%), of which 18/26 (69.2%) and 10/16 (62.5%) were Gram-negative and Gram-positive bacteria, respectively (Table 5).

### 3.4. Factors Associated With UTI

In simple logistic regression analysis, respondents age group 15–24 (COR = 0.338 and 95% CI [0.08, 1.38]), educational status with primary school (COR = 0.343 and 95% CI [0.121, 0.970]) and secondary school (COR = 0.381 and 95% CI [0.187, 0.774]), increased urine frequency (COR = 3.758 95% CI [1.561, 9.048]), PROM (COR = 49.125 95% and CI [15.381, 156.902]), IUFD (COR = 4.383 and 95% CI [0.710, 27.049]), birth weight (COR = 29.467 and 95% CI [10.656, 81.486]), APGAR score (COR = 2.481 95% and CI [0.631, 9.753]), and gestational age at birth (COR = 10.154 and 95% CI [1.644, 62.700]) were found to be candidate (*p* < 0.25) for multivariable logistic regression analysis.

The result of multivariable logistic regression analysis revealed that pregnant women with PROM had a significant association with UTI (*p*=0.001, AOR = 22.828, and 95% CI [5.738, 90.810]) and pregnant women gave underweight (< 2500 g) birth had also a significant association with UTI (*p*=0.001, AOR = 21.48, and 95% CI [6.35, 72.70]) (Table 6).

## 4. Discussion

The prevalence of UTI among pregnant women in the current study (13.6%) is congruent with a study conducted in Iran (13.1%) [10], Tanzania (14.6%) [11], Kenya (15.7%) [12], Johannesburg (16.8%) [13], Dire Dawa (14%) [14], and Addis Ababa (14.9%) [15]. However, the finding of this study is lower than the prevalence of UTI reported from Cameroon (31%) [16], Yemen (30%) [17], and Al Samawa City of Iraq (86%) [18]. In addition, the finding of the current study is higher than the prevalence of UTI reported from Bangladesh (8.9%) [19] and Hawassa Southern Ethiopia (7.8%) [20]. Differences in associated factors, sample size, community social habits, personal standards of hygiene, and education across different studies from one nation to another, as well as between areas of the same country might be the possible reasons for variation in prevalence.

The prevalence of symptomatic bacteriuria (4.5%) in this study was in agreement with the similar study done in Bangladesh (4.4%) [19], lower than the results from Tanzania (17.9%) [11], Kenya (11.4%) [12], Harar (21%) [21], and Cameroon (33.6%) [16] but slightly higher than the results from Bahir Dar (1.9%) [22] and Addis Ababa (3.3%) [15].

The occurrence of asymptomatic UTI (9.1%) in this study was in line with the previous study done in Indonesia (10.2%) [23] but a lower incidence rate was reported in Kenya (4.3%) [12], Bangladesh (4.5%) [19], and Bahir Dar (7.6%) [22]. The difference may be due to geographical variation or may also be due to variation in interpretation of the symptoms.

The predominant isolates in this study were Gram-negative bacteria (61.9%). Different studies conducted in Yemen (73.7%) [17], Bahir Dar (65%) [22], and Indonesia (72%) [23] aligned with this finding but lower than the report from Cameroon (85.4%) [16] and Harar (90.3%) [21].

*E. coli* was the most frequent bacterial isolate (40.5%), which is identical to previous studies in Hawassa (36.2%) [24], Harar (45.2%) [21], systematic review in Ethiopia (41.1%) [25], Yemen (41.5%) [17], Cameroon (43.2%) [16], and Bangladesh (47.2%) [2]. There is a magnified risk of acquiring UTI from *E. coli* since *E. coli* colonizes intestinal tract and trip from the anus to the urethra is a short one, as well as the difficulties of maintaining personal cleanliness during pregnancy, *S. aureus* (14.3%), ranked next to *E. coli* which align with different reports like a systematic review and meta-analysis in Ethiopia 15% [25], Mandya 17.8% [26], and Gonder 10% [27]. In this study, *S. saprophytics* ranked as the third predominant pathogen while *K. Pneumoniae*, *E. faecalis*, and *S. pyogenes* were equally ranked fourth. These findings indicate that even though there are a number of UTI-causing bacterial species, majority of UTI cases are limited to few groups of bacteria.

In this study, most of Gram-negative bacterial isolates were sensitive to cefoxitin (92%), cefepime (92%), amikacin (88.5%), levofloxacin (85.5%), and ciprofloxacin (85.5%). A similar study in Gonder, Ethiopia, showed that Gram-negative bacterial isolates were sensitive to ciprofloxacin (96.3%) [27], and another similar study in India revealed that Gram-negative isolates were sensitive to amikacin (76.7%) and ciprofloxacin (73.3%) [28]. The highest resistance in this study was shown to ampicillin (96.2%) and trimethoprim-sulfamethoxazole (39%), which is consistent with the study in Bahir Dar, Ethiopia. Gram-negative bacteria showed resistance to ampicillin (82.6%) and trimethoprim-sulfamethoxazole (56.5%) [22], and in a similar study in Johannesburg, Gram-negative bacteria showed resistance to ampicillin (70%) [13] and in a study in Russell Hall Hospital Dudley in the United Kingdom Gram-negative bacteria showed resistance to trimethoprim-sulfamethoxazole (36.2%) [29]. *E. coli* in this study was resistant to ampicillin (94.1%) and nitrofurantoin (23.5%) like that of the Cameroon's study with resistance to ampicillin (26.3%) [16] and Harar's study with resistance to ampicillin (50%) and nitrofurantoin (85.7%) [21]. *S. aureus* also showed resistance to trimethoprim-sulfamethoxazole (66.7%) and tetracycline (66.7%), which is similar to Dire Dawa's study with resistance to trimethoprim-sulfamethoxazole (33.3%) [14] and Bahir Dar's study with resistance to trimethoprim-sulfamethoxazole (33.3%) and tetracycline (66.7%) [22].

Multidrug resistant (MDR) was observed in 66.7% of the total bacteria. This finding was higher than the study reported in Addis Ababa, Ethiopia (57.1%) [30] and lower than studies conducted in Dessie, Ethiopia (80.4%) [31], Uyo, Nigeria (100%) [32]. The possible reason for this MDR might be the self-medication history of study participants.

In this study, sociodemographic factors, clinical factors, APGAR score (in the first and fifth minute), IUFD and gestational age at birth had no statistically significant association with maternal UTI; the study conducted in Ghana agreed with this study with respect to APGAR score [33] and the other study done in Addis Ababa similarly reported that gestational age, IUFD, birth weight and APGAR score (in the first and fifth minute) were not associated with maternal UTI. This was in agreement with the current study expect birth weight of the fetus [15]. A study conducted in Chicago elevated risks were observed for exposure to UTI and low birth weight, prematurity, preterm low birth weight, premature labor, hypertension, anemia, and amnionitis, whereas the fetal death were not associated with maternal UTI [34].

In the current study, UTI during pregnancy had significant association with the occurrence of PROM (*p*=0.001, AOR = 22.828, and 95% CI = 5.738, 90.810) and birth weight (*p*=0.001, AOR = 21.48, and 95% CI = 6.35, 72.70). This finding is similar with reports from Dhaka Bangladesh [35], Chicago [34], Pakistan [36], and Addis Ababa [15]. The other study conducted in Australia by Romero et al. showed that nonbacteriuric patients had two thirds the risk of low birth weight infants and half the risk of preterm delivery compared with patients with untreated asymptomatic bacteriuria [37]. The difference in the significance level and odds of occurrence may be attributed to the sample size, the standard of personal hygiene, sociodemographic difference of study subject, and education. A possible mechanism for how UTI in pregnancy affects the risk of premature membrane rupture has been presented.

UTI causes macrophages to release metalloproteinase, which destroys amniotic membranes and increases the likelihood of rupture [38]. The metalloproteinase is zinc dependent proteases whose physiological roles include control of leukocyte migration. They are implicated in tissue destruction I inflammatory and infectious diseases.

### 4.1. Limitation of the Study

This study was done on a small sample size to assess UTI-related fetal outcome.

Due to a lack of resources, we were unable to identify some bacterial isolates into serotypes and were unable to determine the mechanisms of antimicrobial resistance.

Another limitation of the study was that it only included pregnant women in their third trimester; therefore, future studies should include all trimesters to evaluate and compare the prevalence of UTI at different stages of pregnancy.

## 5. Conclusions

The overall prevalence of UTI was 13.6% (95% CI: 10–18) and UTI was found to be significantly associated with fetal outcome. Gram-negative bacteria were predominant causes of UTI. The most common isolates were *E. coli*, *S. saprophyticus*, and *S. aureus.* Furthermore, Gram-positive isolates were highly sensitive to moxifloxacin (100%), ofloxacin (87.5%), and linezolid (87.5%) and Gram-negative isolates were highly sensitive to cefoxitin (92%), cefepime (92%), and ciprofloxacin (85.5%) but resistant to ampicillin (96.2%). So, ampicillin should not be prescribed for treatment of UTI caused by Gram-negative isolates and early detection and management of UTI are crucial to avert adverse fetal outcome.

## Figures and Tables

**Table 1:** Sociodemographic and obstetric characteristics of pregnant women who attended antenatal care at Adare General Hospital, Hawassa, Ethiopia, 2022 (*n* = 308).

Variables		Frequency (*n*)	Percent (%)
Age (in years)	15–24	158	51.3
	25–34	138	44.8
	≥ 35	12	3.9

Marital status	Married	303	98.4
	Divorced	5	1.6

Educational status	Primary school	55	17.9
	Secondary school	160	51.9
	College and above	93	30.2

Occupation	Housewife	183	59.4
	Merchant	55	17.9
	Government employee	63	20.5
	Student	7	2.3

Increased frequency of urine	Yes	27	8.8
	No	281	91.2

Gravidity	Primigravida	56	18.2
	Multigravida	252	81.8

PROM	Yes	22	7.1
	No	286	92.9

IUFD	Yes	5	1.6
	No	303	98.4

Birth weight	Normal	285	92.5
	Under	23	7.5

APGAR score	Normal	297	96.4
	Under	11	3.6

Gestational age at birth	Term	303	98.4
	Preterm	5	1.6

Abbreviations: APGAR = appearance, pulse, grimace, activity, and respiration, IUFD = intrauterine fetal demise, PROM = premature rupture of membrane.

**Table 2:** Types of bacterial isolates from urine culture of pregnant women with UTI (*n* = 42) attending ANC at Adare General Hospital, Hawassa, Ethiopia, from May to August 2022.

Variables	Frequency	Percent (%)
*E. coli*	17	40.5
*S. aureus*	6	14.3
*S. saprophyticus*	4	9.5
*K. pneumoniae*	3	7.1
*E. faecalis*	3	7.1
*S. pyogenes*	3	7.1
*K. oxytoca*	2	4.8
*Citrobacter* spp	2	4.8
*P. mirabilis*	1	2.4
*M. morganii*	1	2.4
Total	42	100

**Table 3:** Antimicrobial susceptibility pattern of Gram-negative bacterial isolates from urine culture of pregnant women attending ANC at Adare General Hospital, Hawassa, Ethiopia, from May to August 2022 (*n* = 26).

Bacterial	Pattern	Antibiotics (%)												
Isolates (N°)		FOX	CZ	TM	SAM	AM	CAZ	CRO	FEP	GM	MEM	SXT	CIP	TOB
*E. coli* (17)	S	15 (88.2)	13 (76.5)	15 (88.2)	11 (64.7)	1 (5.9)	14 (82.4)	15 (88.2)	16 (94.1)	14 (82.4)	16 (94.1)	15 (88.2)	14 (82.4)	16 (94.1)
	I	0 (0)	0 (0)	0 (0)	0 (0)	0 (0)	0 (0)	0 (0)	0 (0)	1 (5.9)	0 (0)	0 (0)	0 (0)	0 (0)
	R	2 (11.8)	4 (23.5)	2 (11.8)	6 (35.3)	16 (94.1)	3 (17.6)	2 (11.8)	1 (5.9)	2 (11.8)	1 (5.9)	2 (11.8)	3 (17.6)	1 (5.9)

*K. pneumoniae* (3)	S	3 (100)	1 (33.3)	1 (33.3)	1 (33.3)	0 (0)	2 (66.7)	1 (33.3)	3 (100)	3 (100)	3 (100)	1 (33.3)	3 (100)	3 (100)
	I	0 (0)	0 (0)	0 (0)	0 (0)	0 (0)	0 (0)	0 (0)	0 (0)	0 (0)	0 (0)	0 (0)	0 (0)	0 (0)
	R	0 (0)	2 (66.7)	2 (66.7)	2 (66.7)	3 (100)	1 (33.3)	2 (66.7)	0 (0)	0 (0)	0 (0)	2 (66.7)	(0) (0)	0 (0)

*K. oxytoca* (2)	S	2 (100)	2 (100)	2 (100)	2 (100)	0 (0)	1 (50)	0 (0)	2 (100)	2 (100)	0 (0)	2 (100)	2 (100)	2 (100)
	I	0 (0)	0 (0)	0 (0)	0 (0)	0 (0)	0 (0)	0 (0)	0 (0)	0 (0)	0 (0)	0 (0)	0 (0)	0 (0)
	R	0 (0)	0 (0)	0 (0)	0 (0)	2 (100)	1 (50)	2 (100)	0 (0)	0 (0)	2 (100)	0 (0)	0 (0)	0 (0)

*Citrobacter* spp (2)	S	2 (100)	2 (100)	2 (100)	1 (50)	0 (0)	0 (0)	1 (50)	1 (50)	2 (100)	1 (50)	0 (0)	2 (100)	2 (100)
	I	0 (0)	0 (0)	0 (0)	0 (0)	0 (0)	0 (0)	0 (0)	0 (0)	0 (0)	0 (0)	0 (0)	0 (0)	0 (0)
	R	0 (0)	0 (0)	0 (0)	1 (50)	2 (100)	2 (100)	1 (50)	1 (50)	0 (0)	1 (50)	2 (100)	0 (0)	0 (0)

*P. mirabilis* (1)	S	1 (100)	1 (100)	1 (100)	1 (100)	0 (0)	0 (0)	1 (100)	1 (100)	0 (0)	0 (0)	0 (0)	1 (100)	1 (100)
	I	0 (0)	0 (0)	0 (0)	0 (0)	0 (0)	1 (100)	0 (0)	0 (0)	0 (0)	0 (0)	0 (0)	0 (0)	0 (0)
	R	0 (0)	0 (0)	0 (0)	0 (0)	1 (100)	0 (0)	0 (0)	0 (0)	1 (100)	1 (100)	1 (100)	0 (0)	0 (0)

*M. morganii* (1)	S	1 (100)	1 (100)	1 (100)	1 (100)	0 (0)	0 (0)	1 (100)	1 (100)	0 (0)	1 (100)	0 (0)	1 (100)	1 (100)
	I	0 (0)	0 (0)	0 (0)	0 (0)	0 (0)	0 (0)	0 (0)	0 (0)	1 (100)	0 (0)	0 (0)	0 (0)	0 (0)
	R	0 (0)	0 (0)	0 (0)	0 (0)	1 (100)	1 (100)	0 (00)	0 (0)	0 (0)	0 (0)	1 (100)	1 (100)	0 (0)

Total (26)	S	24 (92)	20 (76.9)	22 (84.6)	17 (65.4)	1 (3.8)	19 (73.1)	19 (73.1)	24 (92)	22 (84.6)	23 (88.5)	16 (61.5)	23 (88.5)	17 (65.4)
	I	0 (0)	0 (0)	0 (0)	0 (0)	0 (0)	1 (3.8)	0 (0)	0 (0)	2 (7.8)	1 (3.8)	0 (0)	0 (0)	0 (0)
	R	2 (7.7)	6 (23)	4 (15.4)	9 (34.6)	25 (96.2)	6 (23)	7 (30.7)	2 (2.7)	2 (2.7)	2 (2.7)	10 (39)	3 (11.5	9 (34.6)

Abbreviations: AM = ampicillin, CAZ = ceftazidime, CIP = ciprofloxacin, CRO = ceftriaxone, CZ = cefazolin, FEP = cefepime, FOX = cefoxitin, GM = gentamicin, I = intermediate, MEM = meropenem, R = resistant, S = susceptible, SAM = ampicillin/sublactam, SXT = trimethoprim/sulfamethoxazole, TM = piperacillin/tazobactam, TOB = tobramycin.

**Table 4:** Antimicrobial susceptibility pattern of Gram-positive bacterial isolates from urine culture of pregnant women attending ANC at Adare General Hospital, Hawassa, Ethiopia, from May to August 2022 (*n* = 16).

Bacterial	Pattern	Antibiotics (%)														
Isolates (N°)		CRO	MEM	SXT	LEV	AMX	P	CTX	C	ETP	E	LNZ	MXF	OLF	TEL	TE
*S. aureus* (6)	S	5 (83.3)	5 (83.3)	2 (33.3)	4 (66.7)	5 (83.3)	5 (83.3)	4 (66.7)	3 (50)	4 (66.7)	5 (83.3)	6 (100)	6 (100)	5 (83.3)	4 (66.7)	2 (33.3)
	I	0 (0)	0 (0)	0 (0)	0 (0)	0 (0)	0 (0)	0 (0)	0 (0)	0 (0)	0 (0)	0 (0)	0 (0)	0 (0)	0 (0)	0 (0)
	R	1 (16.7)	1 (16.7)	4 (66.7)	2 (33.3)	1 (16.7)	1 (16.7)	2 (33.3)	3 (50)	2 (33.3)	1 (16.7)	0 (0)	0 (0)	1 (16.7)	2 (33.3)	4 (66.7)

*S. saprophyticus* (4)	S	1 (25)	3 (75)	0 (0)	2 (50)	3 (75)	2 (50)	3 (75)	3 (75)	2 (50)	2 (50)	3 (75)	4 (100)	3 (75)	2 (50)	0 (0)
	I	0 (0)	0 (0)	0 (0)	0 (0)	0 (0)	0 (0)	0 (0)	0 (0)	0 (0)	0 (0)	0 (0)	0 (0)	0 (0)	0 (0)	0 (0)
	R	3 (75)	1 (25)	4 (100)	2 (50	1 (25)	2 (50)	1 (25)	1 (25)	2 (50)	2 (50)	1 (25)	0 (0)	1 (25)	2 (50)	4 (100)

*E. faecalis* (3)	S	2 (67)	2 (67)	1 (33)	3 (100)	2 (67)	2 (67)	1 (33)	2 (67)	2 (67)	2 (67)	3 (100)	3 (100)	3 (100)	2 (67)	1 (33)
	I	0 (0)	0 (0)	0 (0)	0 (0)	0 (0)	0 (0)	0 (0)	0 (0)	0 (0)	0 (0)	0 (0)	0 (0)	0 (0)	0 (0)	0 (0)
	R	1 (33)	1 (33)	2 (67)	0 (0)	1 (33)	1 (33)	2 (67)	1 (33)	1 (33)	1 (33)	0 (0)	0 (0)	0 (0)	1 (33)	2 (67)

*S. pyogenes* (3)	S	2 (67)	3 (100)	2 (67)	3 (100)	1 (33)	2 (67)	2 (67)	3 (100)	3 (100)	2 (67)	2 (67)	3 (100)	3 (100)	2 (67)	1 (33)
	I	0 (0)	0 (0)	0 (0)	0 (0)	0 (0)	0 (0)	0 (0)	0 (0)	0 (0)	0 (0)	0 (0)	0 (0)	0 (0)	0 (0)	0 (0)
	R	1 (33)	0 (0)	1 (33)	0 (0)	2 (67)	1 (33)	1 (33)	0 (0)	0 (0)	1 (33)	1 (33)	0 (0)	0 (0)	1 (33)	2 (67)

Total (16)	S	10 (62.5)	12 (75)	5 (31.3)	12 (75)	11 (68.8)	11 (68.8)	10 (62.5)	11 (68.8)	11 (68.8)	11 (68.8)	14 (87.5)	16 (100)	14 (87.5)	10 (62.5)	4 (25)
	I	0 (0)	0 (0)	0 (0)	0 (0)	0 (0)	0 (0)	0 (0)	0 (0)	0 (0)	0 (0)	0 (0)	0 (0)	0 (0)	0 (0)	0 (0)
	R	6 (37.5)	4 (25)	11 (68.8)	4 (25)	5 (31.3)	5 (31.3)	6 (37.5)	5 (31.3)	5 (31.3)	5 (31.3)	2 (12.5)	0 (0)	2 (12.3)	6 (37.5)	12 (75)

Abbreviations: AMX = amoxicillin, C = chloramphenicol, CRO = ceftriaxone, CTX = cefotaxime, E = erythromycin, ETP = ertapenem, I = intermediate, LEV = levofloxacin, LNZ = linezolid, MEM = meropenem, MXF = moxifloxacin, OFL = ofloxacin, P = penicillin, R = resistant, S = susceptible, SXT = trimethoprim/sulfamethoxazole, TE = tetracycline, TEL = telithromycin.

**Table 5:** Multi drug resistance pattern of bacterial isolates from pregnant women with UTI attended ante natal care at AGH, Hawassa, Ethiopia, 2023 (*n* = 42).

Isolates	Frequency (%)				
	Total	R3	R4	≥ R5	MDR
*Gram-negative*	26 (61.9)	11 (61.1)	5 (27.8)	2 (11.1)	18 (69.2)
*E. coli*	17 (65.4)	6 (66.7)	3 (33.3)		9 (50)
*K. pneumoniae*	3 (11.5)	2 (66.7)		1 (33.3)	3 (16.7)
*K. oxytoca*	2 (7.7)	2 (100)			2 (11.1)
*Citrobacter* spp	2 (7.7)		1 (50)	1 (50)	2 (11.1)
*P. mirabilis*	1 (3.8)		1 (100)		1 (5.6)
*M. morganii*	1 (3.8)	1 (100)			1 (5.6)

*Gram-positive*	16 (38.1)	7 (70)	3 (30)		10 (56.3)
*S. aureus*	6 (0.38	3 (100)			3 (30)
*S. saprophyticus*	4 (25)	1 (25)	3 (75)		4 (40)
*E. faecalis*	3 (18.8)	2 (100)			2 (20)
*S. pyogenes*	3 (18.8)	1 (100)			1 (10)

Total	42 (100)	18 (64.3)	8 (28.6)	2 (7.1)	28 (66.7)

*Note:* MDR = resistance for three or more classes/sub classes of antibiotics, R3 = resistance to three drugs, R4 = resistance to four drugs, R5 = resistance to five and more drugs.

**Table 6:** Simple and multivariable logistic regression analysis of factors associated with UTIs among pregnant women attending ANC at Adare General Hospital, Hawassa, Ethiopia, from May to August 2022.

Variables		UTI		COR (95% CI)	*p* value	AOR (95% CI)	*p* value
		Yes (%)	No (%)				
Age (in years)	15–24	16 (10.1)	142 (89.9)	0.338 (0.08, 1.38)	0.130	0.201 (0.036, 1.124)	0.068
	25–34	23 (16.7)	115 (83.3)	0.600 (0.151, 2.388)	0.469	0.270 (0.050, 1.454)	0.127
	≥ 35	3 (25.0)	9 (75.0)	1			

Educational status	Primary school	5 (9.1)	50 (90.9)	0.343 (0.121, 0.970)	0.044	0.424 (0.113, 1.592)	0.204
	Secondary school	16 (10.0)	144 (90.0)	0.381 (0.187, 0.774)	0.008	0.398 (0.151, 1.050)	0.063
	College and above	21 (22.6)	72 (77.4)	1			

Occupation	Housewife	18 (9.8)	165 (90.2)	0.655 (0.075, 5.745)	0.702		
	Merchant	8 (14.5)	47 (85.5)	1.021 (0.108, 9.649)	0.985		
	Government	15 (23.8)	48 (76.2)	1.875 (0.209, 16.837)	0.575		
	Student	1 (14.3)	6 (85.7)	1			

Increased urine frequency	Yes	9 (33.3)	18 (66.7)	3.758 (1.561, 9.048)	0.003	2.156 (0.592, 7.857)	0.244
	No	33 (11.7)	248 (88.3)	1			

Gravidity	Primigravida	8 (14.3)	48 (85.7)	1.069 (0.465, 2.454)	0.876		
	Multigravida	34 (13.5)	218 (86.5)	1			

PROM	Yes	18 (81.8%)	4 (18.2%)	49.125 (15.381, 156.902)	0.001	22.828 (5.738, 90.810)	0.001^∗^
	No	24 (8.4%)	262 (91.6%)	1		1	

IUFD	Yes	2 (40%)	3 (60%)	4.383 (0.710, 27.049)	0.111	8.47 (0.873, 82.145)	0.065
	No	40 (13.2%)	263 (86.8%)	1		1	

Birth weight	Normal	25 (8.8%)	260 (91.2%)	1	0.001	1	0.001^∗^
	Under	17 (73.9%)	6 (26.1%)	29.467 (10.656, 81.486)		21.48 (6.35, 72.70]	

APGAR score	Normal	39 (13.1%)	258 (86.9%)	1	0.193	1	0.964
	Low	3 (27.3%)	8 (72.7%)	2.481 (0.631, 9.753)		1.053 (0.112, 9.898)	

Gestational age at birth	Term	39 (12.9%)	264 (87.1%)	1	0.013	1	0.426
	Preterm	3 (60%)	2 (40%)	10.154 (1.644, 62.700)		3.755 (0.145, 97.331)	

## Data Availability

The data that support the findings of this study are available from the corresponding author upon reasonable request.
